# Characterization of H_2_O_2_-Induced Alterations in Global Transcription of mRNA and lncRNA

**DOI:** 10.3390/antiox11030495

**Published:** 2022-03-03

**Authors:** Shihua Liu, Ya Qiu, Rong Xiang, Peng Huang

**Affiliations:** 1 State Key Laboratory of Oncology in South China, Collaborative Innovation Center for Cancer Medicine, Sun Yat-sen University Cancer Center, Guangzhou 510060, China; liush13@sysucc.org.cn (S.L.); qiuya@sysucc.org.cn (Y.Q.); xiangrong@sysucc.org.cn (R.X.); 2 Center for Cancer Metabolism and Intervention Research, Sun Yat-sen University Cancer Center, Guangzhou 510060, China

**Keywords:** hydrogen peroxide, reactive oxygen species, transcription factors, lncRNA, PROMO, TRRUST

## Abstract

Hydrogen peroxide (H_2_O_2_) is an important reactive oxygen species that plays a major role in redox signaling. Although H_2_O_2_ is known to regulate gene expression and affect multiple cellular processes, the characteristics and mechanisms of such transcriptional regulation remain to be defined. In this study, we utilized transcriptome sequencing to determine the global changes of mRNA and lncRNA transcripts induced by H_2_O_2_ in human pancreatic normal epithelial (HPNE) and pancreatic cancer (PANC-1) cells. Promoter analysis using PROMO and TRRUST revealed that mRNAs and lncRNAs largely shared the same sets of transcription factors in response to ROS stress. Interestingly, promoters of the upregulated genes were similar to those of the downregulated transcripts, suggesting that the H_2_O_2_-responding promoters are conserved but they alone do not determine the levels of transcriptional outputs. We also found that H_2_O_2_ induced significant changes in molecules involved in the pathways of RNA metabolism, processing, and transport. Detailed analyses further revealed a significant difference between pancreatic cancer and noncancer cells in their response to H_2_O_2_ stress, especially in the transcription of genes involved in cell-cycle regulation and DNA repair. Our study provides new insights into RNA transcriptional regulation upon ROS stress in cancer and normal cells.

## 1. Introduction

Reactive oxygen species (ROS) encompass a group of chemically reactive molecules derived from molecular oxygen via reduction–oxidation (redox) reactions or electronic excitation [1]. Due to the increased metabolic demand for continuous proliferation, cancer cells often generate more ROS compared with normal cells [2]. A moderately elevated level of ROS may promote tumor cell proliferation, while extremely high concentrations of ROS can cause oxidative damage to proteins and nucleic acids, leading to cell death [3]. Therefore, controlling the balance between ROS and antioxidants is essential for cellular homeostasis, and targeting ROS metabolism could be an effective strategy to inhibit cancer cell growth as a therapeutic approach [4]. The current redox-modulating strategies for disease treatment exhibit both advantages and limitations [5]. The fact that the use of antioxidants for treatment of diseases with oxidative stress often leads to limited success or even disappointing results suggests that a better understanding of redox signaling and its biological impact is required to provide a mechanistic basis for developing more effective therapeutic strategies.

Redox balance plays a critical role in maintaining the biologic process under homeostatic conditions [6,7]. The cellular redox system may modify the functions of proteins through regulating their expression, causing changes in posttranslational modifications, and/or affect their stabilities [8]. At the synthesis level, expression of signaling proteins can be tightly controlled through the rate of gene transcription [9,10]. Some important transcription factors such as NF-κB [11,12], AP-1 [13], Nrf2 [14,15], and HIF [16,17] can be significantly affected by the redox system. In many cases, thiol oxidation of these proteins attenuates their DNA-binding activities and, thus, affects their function as transcriptional factors [8]. Conversely, the activation of transcriptional factors such as Nrf2 and HIF1 leads to the expression of key molecules that play critical roles in cellular metabolism and cell survival through their functions in protecting against oxidative damage induced by acute injury, hyperoxia, nitrosative stress, ER stress, and exogenous prooxidative agents [1]. Redox signaling in transcription regulation is an essential part of these regulatory processes.

Among the various species of ROS, hydrogen peroxide (H_2_O_2_) has a relatively long half-life and is able to pass through biological membranes [18,19]. These special physiochemical properties enable H_2_O_2_ to function as an “ideal” messenger for redox signaling within a cell and between different cells [20]. As such, there have been multiple studies on how H_2_O_2_ affects gene transcription, often focusing on the expression of mRNA that encodes proteins. Very few studies on the impact of H_2_O_2_ on the expression of noncoding regions of the genome such as long noncoding RNA (lncRNA) have been reported, since the noncoding regions were initially considered as nonfunctional sequences in the genome. However, recent studies have shown that the transcribed RNA from the noncoding region can regulate the expression of coding genes in a variety of ways and may participate in cancer development [21]. LncRNAs play a broad regulatory role in various physiological and pathological processes, and their important role in tumorigenesis and development is one of the hotspots and focuses of current oncology research [22]. Recent studies revealed that lncRNA could play a role in response to oxidative stress, suggesting that lncRNA may be involved in the redox signaling [23,24,25]. However, the regulation of lncRNA transcription in response to ROS stress largely remains unknown. This study used transcriptome sequencing and bioinformatic analyses to investigate the changes in global transcription of mRNA and lncRNA in response to H_2_O_2_ stress and explored the regulatory mechanisms with respect to transcription promoter characteristics.

## 2. Materials and Methods

### 2.1. Cell Culture

The immortalized human pancreatic normal epithelial cell line (hTERT-HPNE) and pancreatic cancer cell line (PANC-1) were from American Type Culture Collection (ATCC; Manassas, VA, USA). Cells were maintained in Dulbecco’s modified Eagle’s medium (DMEM) supplemented with 10% FBS (Corning, New York, NY, USA) at 37 °C in a 5% CO_2_ cell culture incubator as described previously [26]. These two cell lines were authenticated by short tandem repeat (STR) DNA profiling and verified to be mycoplasma-free by periodical testing.

### 2.2. Cell Viability Analysis

Two methods were used to evaluate cell viability. MTS analysis was used to measure the impact of H_2_O_2_ on cell proliferation. Cells were seeded into 96-well plates (3000 cells/well in 100 μL medium) overnight to allow cell attachment, and then 100 μL of medium containing various concentrations of H_2_O_2_ was added. After incubation with H_2_O_2_ in the indicated time periods, 3-(4,5-dimethylthiazol-2-yl)-5-(3-carboxymethoxyphenyl)-2-(4-sulfophenyl)-2*H*-tetrazolium (MTS regent) was added to each well (20 μL/well) and incubated at 37 °C for 3 h. Absorbance at a wavelength of 490 nm was measured using a MultiSkan plate reader. For apoptosis analysis, a FITC–annexin V Apoptosis Detection Kit (BD Pharmingen™, San Diego, CA, USA, BD556547) was used to stain apoptotic cells according to the manufacturer’s instructions for flow cytometry. The results were analyzed using the CytExpert (Beckman Coulter, Brea, CA, USA) software.

### 2.3. Hydrogen Peroxide Treatment and RNA Extraction

HPNE and PANC-1 cell lines were treated with hydrogen peroxide (H_2_O_2_, Sigma, Saint Louis, MO, USA) 0.3 mM and 1.0 mM for 12 h, respectively. These concentrations were selected on the basis of the cytotoxicity test results. The selective concentrations did not cause significant toxicity under the H_2_O_2_ exposure condition in the respective cell lines. Total RNA was isolated using TRIzol (Invitrogen, Carlsbad, CA, USA) according to the manufacturer’s instructions.

### 2.4. Sample Qualification and Quantification

Total RNA was qualified and quantified as follows: (1) the RNA sample was firstly analyzed using 1% agarose gel electrophoresis to check for potential contamination and degradation; (2) RNA purity and concentration were then examined using a NanoPhotometer^®^ spectrophotometer; (3) RNA integrity and quantify were finally measured using a RNA Nano 6000 Assay Kit (Agilent, Santa Clara, CA, USA) before analysis using the Bioanalyzer 2100 system. The standard RIN value of our RNA samples was set as RIN^e^ ≥ 6.5. The RIN values for the specific samples were as follows: HPNE/Ctrl, 9.9; HPNE/H_2_O_2_, 9.2; PANC-1/Ctrl, 10; PANC-1/H_2_O_2_, 10.

### 2.5. Library Preparation and Sequencing

The ribosomal RNA was depleted from total RNA and RNA was then fragmented into 250–300 bp fragments. The RNA library fragments were purified with AMPure XP system. After library construction, the concentration of the library was measured using a Qubit^®^ flurometer (Thermo Fisher, Waltham, MA, USA) and adjusted to 1 ng/μL. An Agilent 2100 Bioanalyzer (Agilent, Santa Clara, CA, USA) was deployed to examine the insert size of the acquired library. Samples were subjected to Illumina sequencing; the lncRNA-seq used PE150 (paired-end 150 nt) sequencing, yielding 12 GB of raw data with approximately 80 million reads.

Raw reads of FASTQ format were first processed through in-house perl scripts. In this step, clean reads were obtained by removing the following reads: (1) reads with 5′ adaptor; (2) reads without 3′ adaptor or insert sequence; (3) reads with more than 10% N; (4) reads with more than 50% nucleotides with Qphred ≤ 20; (5) reads with poly A/T/G/C. Adaptor trimming for the removal of adapter sequences from the 3′ ends of reads was also performed.

### 2.6. Identification of lncRNA

Clean reads for each sample were first mapped to the reference genome Ensembl Release 104 (https://asia.ensembl.org/index.html, accessed on 31 May 2021) with the software HISAT2. Read alignment results were transferred to the program StringTie for transcript assembly. lncRNAs were identified from the assembled transcripts following four steps: (1) removal of low expressed transcripts with FPKM < 0.5; (2) removal of short transcripts <200 bp and <2 exons; (3) removal of the transcripts with protein-coding capability using CNCI, Pfam, and CPC2 databases; (4) removal of the transcripts mapped within the 1 kb flanking regions of annotated genes using Cuffcompare. Novel lncRNAs were named following the rules of the HGNC (HUGO Gene Nomenclature Committee).

### 2.7. Quantification and Differential Expression Analysis

Quantification of the transcripts and genes was performed using StringTie software, and fragments per kilobase of transcript per million mapped reads (FPKM) values were obtained. Cuffdiff or edgeR was used for differential expression analysis. The resulting *p*-values were adjusted using the Benjamini and Hochberg’s approach for controlling the false discovery rate. Genes with |log_2_ (fold change)| > 0 and *p*_adj_ < 0.05 were assigned as differentially expressed.

### 2.8. lncRNA Target Gene Prediction

The *cis*-acting target gene prediction strategy was used to predict the target gene of lncRNAs. According to the theory of *cis*-acting regulatory elements, the protein-coding genes located within 100 kb of the lncRNA were selected as potential *cis*-acting targets.

### 2.9. Transcription Factor Prediction

Promoter sequences 2000 bp in front of initial transcription were downloaded from the UCSC database (UCSC Genome Browser on Human Dec. 2013 (GRCh38/hg38) Assembly). Transcription factors were predicted using PROMO (version 8.3 of TRANSFAC, http://alggen.lsi.upc.es/cgi-bin/promo_v3/promo/promoinit.cgi?dirDB=TF_8.3, accessed on 30 December 2021). Considering factors and sites were only human, the maximum matrix dissimilarity rate was set to 0. The TRRUST (Transcriptional Regulatory Relationships Unraveled by Sentence-Based Text Mining) software (version 2, https://www.grnpedia.org/trrust/, accessed on 30 December 2021) was used to find key regulators for query genes.

### 2.10. Quantitative Reverse-Transcription Polymerase Chain Reaction

RNA was reverse-transcribed using the Primer Script RT reagent Kit with a gDNA Eraser (Takara BIO INC, Kusatsu, Shiga, Japan). Real-time PCR was performed using the SYBR Premix Ex Taq RNase H+ kit (Takara) and analyzed using the ROCHE 480 384 well. The samples were first incubated for 5 min at 95 °C, followed by 40 cycles of 10 s at 95 °C and 30 s at 60 °C. The results were calculated (formula: 2^−(Ct target − Ct ACTB)^) and matched to the control samples. The primer sequences are listed in Table S3, as produced by Sangon Biotech (Shanghai, China).

### 2.11. Deposit of Transcriptome Data in Public Database

The transcriptome sequence data were deposited in the Gene Expression Omnibus (GEO) database: http://www.ncbi.nlm.nih.gov/geo/ under accession number GSE196284.

## 3. Results

### 3.1. Global Changes in mRNA and lncRNA Transcripts in Response to H_2_O_2_ Stress in Normal Pancreatic Epithelial and Pancreatic Cancer Cells

**Figure 1:**
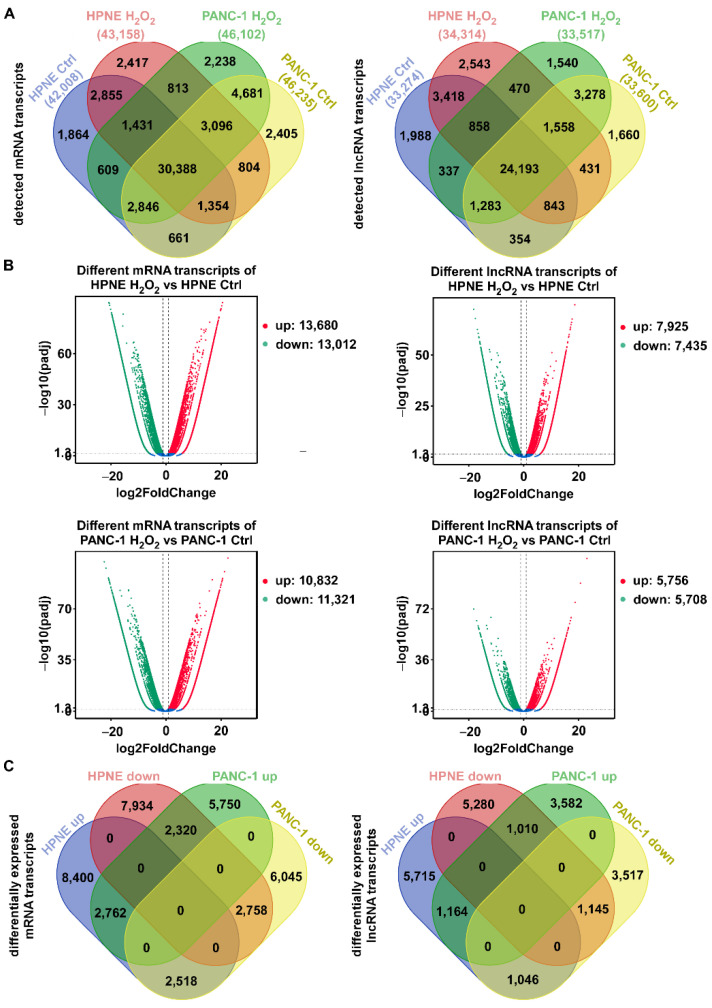
Global changes in mRNA and lncRNA transcripts in response to H_2_O_2_ stress in HPNE and PANC-1 cells. (**A**) Venn diagrams showing mRNA (**left**) and lncRNA (**right**) transcripts detected in control HPNE cells, H_2_O_2_-treated HPNE cells, control PANC-1 cells, and H_2_O_2_-treated PANC-1 cells. The overlapping regions show the co-expression of transcripts in the indicated samples. (**B**) Volcano plots showing the differentially expressed mRNA (**left**) and lncRNA (**right**) transcripts in the control and H_2_O_2_-treated HPNE cells (**top**) and PANC-1 cells (**bottom**). Red points: log_2_ (fold change) > 1 and *p*_adj_ < 0.05; green points: log_2_ (fold change) < −1 and *p*_adj_ < 0.05. (**C**) Venn diagrams showing the up- and downregulated differentially expressed mRNA (**left**) and lncRNA (**right**) transcripts with |log_2_ (fold change) > 1 and *p*_adj_ < 0.05 in both cell lines.

We utilized the immortalized human pancreatic normal epithelial cell line hTERT-HPNE and pancreatic cancer cell line PANC-1 to investigate their transcriptional response to ROS stress induced by H_2_O_2_ treatment. Due to the different sensitivity of the two cell lines to H_2_O_2_, we first determined their respective subtoxic concentrations to ensure that the cells were exposed to comparable subtoxic concentrations in the subsequent experiments. As shown in Figure S1 (Supplementary Materials), the comparable subtoxic concentrations of H_2_O_2_ (12 h treatment) for HPNE and PANC-1 cells were 0.3 mM and 1.0 mM, respectively. After cells were treated with or without H_2_O_2_ for 12 h, RNA was extracted from the control and H_2_O_2_-treated cells for transcriptome sequencing (GSE196284). As shown in Figure 1A, there were 42,008, 43,158, 46,235, and 46,102 mRNA transcripts detected in the HPNE control, H_2_O_2_-treated HPNE, PANC-1 control, and H_2_O_2_-treated PANC-1 cells, respectively. The numbers of lncRNA transcripts for these four samples were 33,274, 34,314, 33,600, and 33,517, respectively. The *R*^2^ values of Pearson correlation between the transcripts in the control samples and those in the H_2_O_2_-treated samples were 0.992 and 0.974 for HPNE and PANC-1 cells, respectively. Among approximately 44,000 detectable mRNA transcripts, 30,388 (69%) mRNAs were commonly detected in all four samples. Similarly, among approximately 34,000 detectable lncRNA transcripts, 24,193 (71%) lncRNAs were detected in all four samples, suggesting that the majority of the transcripts were commonly expressed, although their levels of expression might differ between cell types and could be affected by H_2_O_2_ treatment. The detailed distributions of these RNA transcripts in HPNE and PANC-1 cells with or without H_2_O_2_ exposure are illustrated as Venn diagrams in Figure 1A.

Compared with control cells, H_2_O_2_ treatment induced an upregulation of 13,680 mRNA transcripts and a downregulation of 13,012 mRNAs in HPNE cells, using fold change > 2 and *p* < 0.05 as the cutoff level (Figure 1B). In PANC-1 cells; H_2_O_2_ caused an upregulation of 10,832 mRNA transcripts and a downregulation of 11,321 mRNAs (Figure 1B, left). The numbers of differentially expressed lncRNA transcripts in response to H_2_O_2_ treatment were about half of that of mRNA transcripts (Figure 1B, right). Further analysis showed that, among the differentially expressed RNA transcripts, there were 2762 mRNA and 1164 lncRNA transcripts commonly upregulated in both cell lines, and 2758 mRNA and 1145 lncRNA transcripts commonly downregulated in both cell lines (Figure 1C). Compared with the large number of differentially expressed RNA transcripts in a single cell line, the relatively small number of H_2_O_2_-induced transcripts common in both cell lines suggests that HPNE cells and PANC-1 cells seemed to have different regulatory mechanisms in response to ROS stress.

The transcriptome sequencing results showed that the expression of some well-known oxidative stress responsive genes (TXNRD1, TXNRD2, HMOX1, HMOX2) and ROS stress-responsive lncRNAs (MALAT1, LUCAT1, ODRUL, LINC01619, LINC00963, BDNF-AS) were upregulated by H_2_O_2_ in both cell lines (Figure S2). Of note, TXNRD1, TXNRD2, HMOX1, and HMOX2 are targeted genes of the NRF2/Keap1/ARE signaling pathway. Similarly, lncRNAs MALAT1, LUCAT1, and ODRUL could also be induced by the NRF2/Keap1/ARE pathway [23]. Thus, the upregulation of these mRNAs and lncRNAs seemed to reflect a strong activation of the Nrf2 signaling pathway in respond to H_2_O_2_ stress.

### 3.2. Comparison of Changes in Signal Transduction Pathways Induced by H_2_O_2_ in Pancreatic Epithelial Cells and Pancreatic Cancer Cells

**Figure 2:**
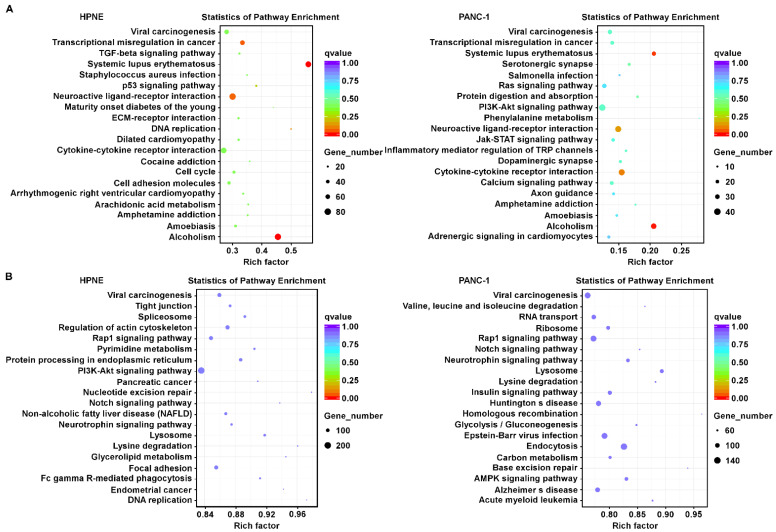
KEGG pathway enrichment analysis of differentially expressed genes and the differentially expressed lncRNA-colocalized genes. (**A**) Top 20 pathways revealed by KEGG enrichment analysis of differentially expressed genes induced by H_2_O_2_ treatment in HPNE cells (**left**) and PANC-1 cells (**right**). The analysis was performed using KOBAS software. (**B**) Top 20 pathways revealed by KEGG enrichment analysis of genes whose DNA loci were colocalized with the differentially expressed lncRNAs (<100 kb) after H_2_O_2_ treatment. In each diagram, the vertical axis shows the names of the pathways, and the horizontal axis represents the enrichment factor (the proportion of candidate gene relative to the background genes). The size of the dot represents the number of differentially expressed genes in the respective pathway, and the color of the dot indicates the Q value ranges.

To investigate the alterations of signal transduction pathways induced by H_2_O_2_ in HPNE and PANC-1 cells, KEGG pathway enrichment analysis was utilized to reveal the altered pathways according to the changes in gene transcriptions. As shown in Figure 2A, the H_2_O_2_-induced changes in RNA transcripts commonly enriched in both cells cell lines were pathways involved in viral carcinogenesis, transcriptional misregulation in cancer, systemic lupus erythematosus, neuroactive ligand–receptor interaction, cytokine–cytokine receptor interaction, amphetamine addiction, amoebiasis, and alcoholism (Figure 2A). Since the functions of most lncRNAs still remain unclear, we searched their colocalized genes as possible *cis*-regulated targets. KEGG pathway enrichment analysis revealed that the H_2_O_2_-induced lncRNAs colocalized with genes that were enriched in pathways involved in viral carcinogenesis, Rap1 signaling, notch signaling, neurotrophin signaling, lysosome, and lysine degradation in both cell lines (Figure 2B). Compared with the differentially expressed genes (mRNAs), the differentially expressed lncRNAs seemed more enriched with potential *cis*-regulated genes involved in metabolism such as pyrimidine metabolism, protein processing in endoplasmic reticulum, lysine degradation, glycerolipid metabolism, valine, leucine, and isoleucine degradation, glycolysis/gluconeogenesis, and carbon metabolism (Figure 2). These data suggest a potential role of lncRNA in regulating cell metabolism upon ROS stress.

We then utilized quantitative reverse transcription PCR (qRT-PCR) in biological triplicates to confirm our findings from the global transcriptomic analysis of gene expression changes induced by H_2_O_2_. Since the signaling of “transcriptional misregulation in cancer” (hsa05202) exhibited consistent changes in both cells by transcriptome sequence (Figure S3A), we measured the expression of the enriched genes in this KEGG pathway. As shown in Figure S3B, the mRNA expression of the enriched genes in “transcriptional misregulation in cancer” was highly consistent with the results of global RNA-seq. Notably, H3 clustered histone 3 members were globally decreased in HPNE and PANC-1 cells (Figure S3B). We also measured the expression of genes associated with this signal cluster in cells treated with H_2_O_2_ treatment for 1 h and 12 h, and we obtained consistent results (Figure S4).

**Figure 3:**
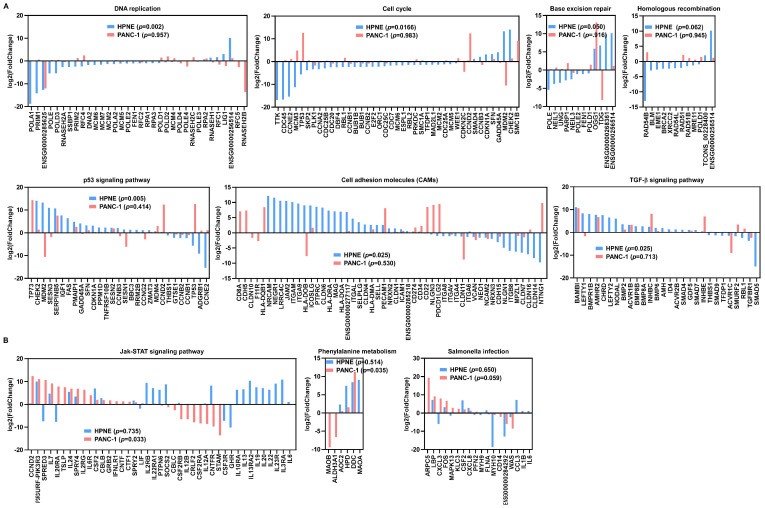
Distribution of differentially expressed genes in the H_2_O_2_-induced pathways identified by KEGG pathway enrichment analysis. (**A**) Expression of enriched genes in the indicated pathways associated with DNA replication, cell cycle, homologous recombination, base excision repair, p53 signaling, cell adhesion molecules (CAMs), and TGF-β signaling in HPNE cells. (**B**) Expression of enriched genes in Jak/STAT signaling pathway, phenylalanine metabolism, and salmonella infection, which changed significantly in PANC-1 cells.

Interestingly, the result of KEGG enrichment analysis revealed some significant differences between normal pancreatic epithelial cells (HPNE) and pancreatic cancer cells (PANC-1) in response to H_2_O_2_ exposure. HPNE cells showed gene expression enrichment in pathways of DNA replication, cell cycle, homologous recombination, base excision repair, and p53-related pathway (Figure 3A). In contrast, H_2_O_2_ failed to induce the p53 signaling pathway and genes involved cell-cycle arrest in PANC-1 cells (Figure 3A). Moreover, cell adhesion molecules and the TGF-β signaling pathway were only activated in HPNE cells (Figure 3A), whereas genes involved in the Jak/STAT signaling pathway, phenylalanine metabolism, and salmonella infection were activated by H_2_O_2_ in PANC-1 cells (Figure 3B).

### 3.3. Alterations of mRNA and lncRNA Transcripts Induced by H_2_O_2_ Largely Shared the Same Promoters

**Figure 4:**
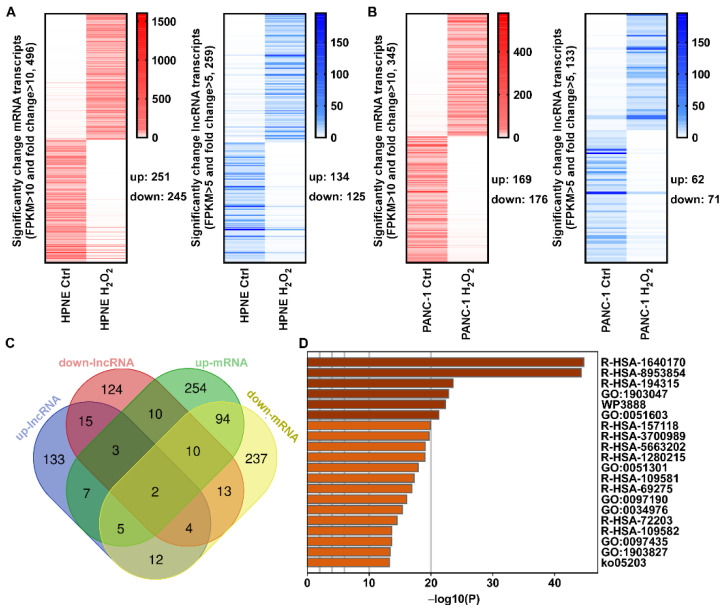
Heatmap, Venn diagram, and KEGG pathway enrichment of significantly altered expression of RNA transcripts for promoter analysis. (**A**,**B**) Heatmap diagrams showed the significantly changed mRNA (fold change >10 and FPKM > 10) and lncRNA (fold change > 5 and FPKM > 5) transcripts upon H_2_O_2_ treatment in HPNE cells (**A**) and PANC-1 cells (**B**). (**C**) Gene loci of significantly changed transcripts under H_2_O_2_ treatment in both cell lines. The differentially expressed RNA transcripts in the four groups (two cell lines treated with or without H_2_O_2_ as indicated) were analyzed by Venn diagram. The overlapping region for all samples contained two genes (PKM and PSMA6). (**D**) Histogram of the top 20 GO enrichment terms for significantly changed transcripts upon H_2_O_2_ treatment in both cell lines, generated using the Metascape tool. The detailed description of terms is shown in Table 1.

To explore the transcription factors that regulated mRNA transcripts after H_2_O_2_ treatment, we first identified significantly changed mRNA transcripts that were differentially expressed in response to H_2_O_2_ with substantial fold change (FC > 10, FPKM > 10). There were 251 upregulated and 245 downregulated mRNA transcripts identified in the HPNE cell line (Figure 4A), and 169 upregulated and 176 downregulated mRNA transcripts in the PANC-1 cell line (Figure 4B). Similarly, lncRNA transcripts with a substantial fold change (FC > 5, FPKM > 5) induced by H_2_O_2_ were also identified. There were 134 upregulated and 125 downregulated lncRNA transcripts identified in the HPNE cell line (Figure 4A), and 62 upregulated and 71 downregulated lncRNA transcripts in PANC-1 cells (Figure 4B). Many significantly changed lncRNA and mRNA transcripts shared the same gene ID, and some of them had the same expressed trend after H_2_O_2_ treatment. However, different transcripts in the same gene locus with different expressed trend also occurred in a small number of cases (Figure 4C). GO Pathway enrichment analysis using Metascape showed that the significantly changed RNA transcripts were enriched in pathways associated with cell-cycle regulation and RNA metabolism (Figure 4D and Table 1), consistent with the results shown in Figure 2.

**Figure 5:**
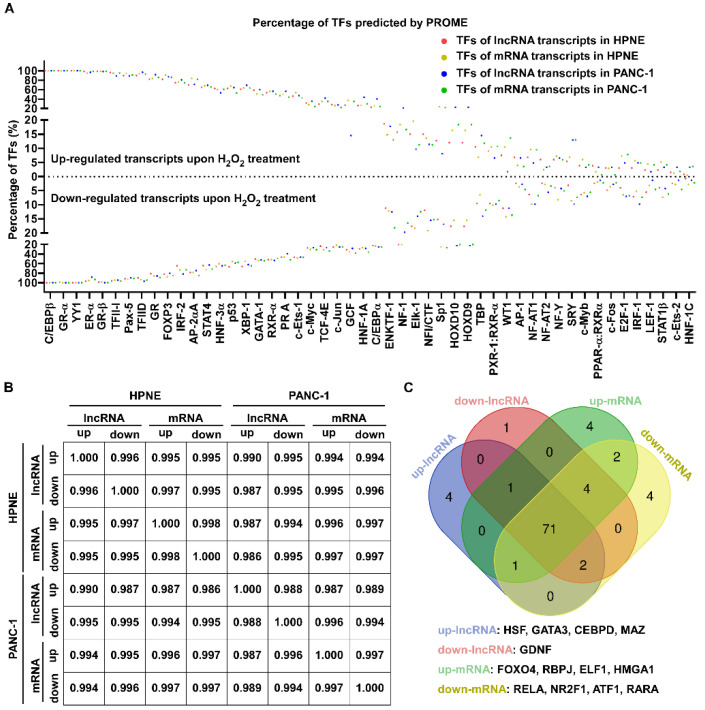
Transcription factors responding to H_2_O_2_ stress in HPNE and PANC-1 cells revealed by PROMO analysis. (**A**) Transcription factors of significantly changed lncRNA and mRNA transcripts promoters were predicted by PROMO analysis, and the percentages of transcription factors distributed in promoters of the up- and downregulated lncRNA and mRNA transcripts after H_2_O_2_ treatment of HPNE and PANC-1 cells are shown by dot plot. The dots below the dotted line represent the promoters of downregulated transcripts, while the top dots represent the promoters of upregulated transcripts. (**B**) The correlation of transcription factors between the up- and downregulated lncRNA and mRNA transcripts after H_2_O_2_ treatment in HPNE and PANC-1 cells. (**C**) Venn diagram showing the transcription factors not shown in Figure 4A. The transcription factors observed only in one type of transcriptional change are shown under the Venn diagram.

To analyze the transcription factors for the mRNA and lncRNA transcripts induced by H_2_O_2_, potential promoter regions approximately 2000 bp in front of transcriptional initiation sites of the significantly changed transcripts were downloaded from the UCSC database, and PROMO (version 8.3 of TRANSFAC) was used to predict transcription factors. The results indicated that the promoters of up- or downregulated transcripts could be recognized by the same transcription factors (Figure 5A and Table S1). Moreover, the percentage of transcription factors for the upregulated RNA transcript promoters was similar to that for the downregulated transcripts (Figure 5A). Correlative analysis indicated that, regardless of whether the lncRNAs or mRNAs were upregulated or downregulated in HPNE or PANC-1 cells, the H_2_O_2_-induced changes of transcripts consistently had DNA elements in their promoters recognized by the same transcription factors (Figure 5B). Among the 94 transcription factors identified in both cell lines, we found that 71 of them (75%) were common in up- or downregulated lncRNA and mRNA transcripts, although a few of the transcription factors occurred only in up- or downregulated lncRNA and mRNA transcripts (Figure 5C).

**Figure 6:**
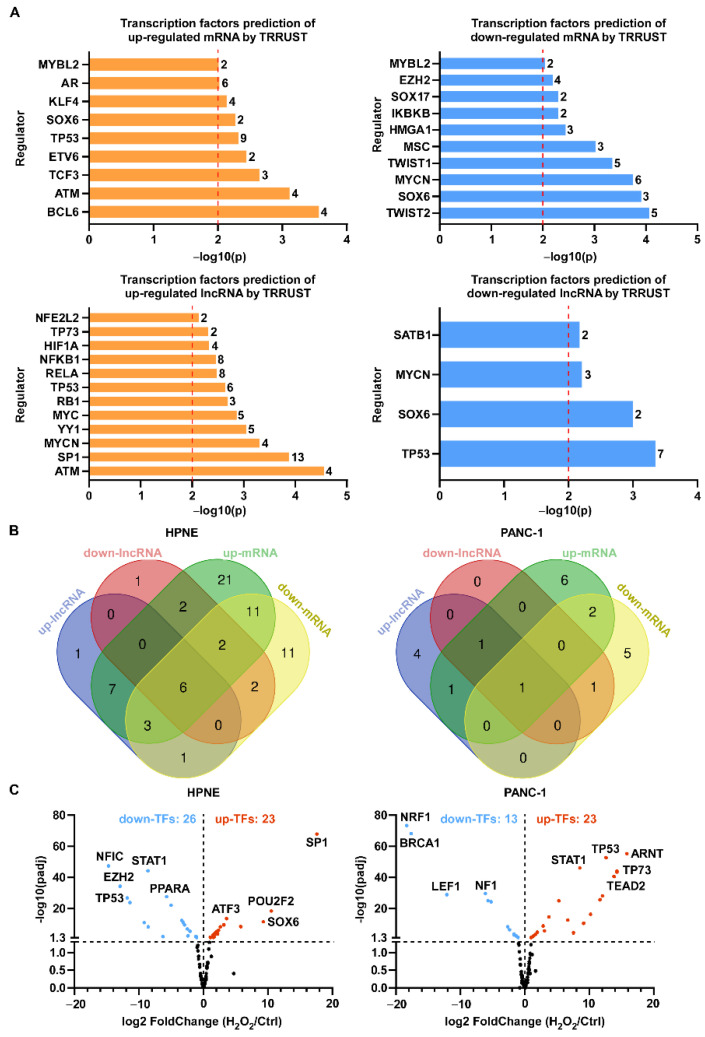
Transcription factors revealed by TRRUST analysis in HPNE and PANC-1 cells treated with H_2_O_2_. (**A**) Top transcription factors (−log_10_(*p*) > 2) of up- and downregulated lncRNA (**top**) and mRNA (**bottom**) transcripts upon H_2_O_2_ treatment in both cell lines were analyzed by TRRUST, and the counts of transcription factors are presented after the column. (**B**) Venn diagram showing all transcription factors predicted by TRRUST in HPNE (**left**) and PANC-1 (**right**) cell lines; the transcription factors are listed in Table 2 for HPNE cells and Table 3 for PANC-1 cells. (**C**) Volcano plots showing the mRNA levels of transcription factors predicted by PROMO and TRRUST upon H_2_O_2_ treatment in HPNE cells (**left**) and PANC-1 cells (**right**). Red dots represent the upregulated transcription factors (log_2_ (fold change) > 1 and *p*_adj_ < 0.05) upon H_2_O_2_ treatment, while blue dots represent the downregulated transcription factors (log_2_ (fold change) < −1 and *p*_adj_ < 0.05).

Since most significantly changed lncRNA transcripts were extronic, we used gene symbols to represent lncRNA transcripts in another analysis. TRRUST (Transcriptional Regulatory Relationships Unraveled by Sentence-Based Text Mining) was used to find the key regulators for significantly changed RNA transcripts (Table S2), and the top transcription factors (*p* < 0.01) are shown in Figure 6A. Consistent with previous reports about mRNA transcription, redox-associated regulators such as NFE2-like bZIP transcription factor 2 (NFE2L2), hypoxia-inducible factor 1 subunit alpha (HIF1α), and nuclear factor kappa B subunit 1 (NFKB1) were found to regulate lncRNA expression under ROS stress in HPNE cell line (Figure 6A,B, Table 2). Some transcription factors predicted by TRRUST were also found to regulate both up- and downregulated transcripts. These included signal transducer and activator of transcription 3 (STAT3), NF-κB subunit RELA, tumor protein p53 (TP53), NFKB1, Sp1 transcription factor (SP1), and estrogen receptor 1 (ESR1) (Figure 6B, Table 2 and Table 3).

We next explored the expression levels of transcription factors predicted by PROMO and TRRUST. There were 26 downregulated and 23 upregulated transcription factors in HPNE, and 13 downregulated and 23 upregulated transcription factors in PANC-1 upon H_2_O_2_ treatment (Figure 6C). For potential prediction of RNA transcript regulation by H_2_O_2_, there seemed to be no direct correlation between the expression of transcription factors and differentially expressed RNA transcripts. For instance, SP1 was significantly upregulated upon H_2_O_2_ treatment, but TRRUST predicted it could recognize promoters of upregulated and downregulated lncRNA and mRNA transcripts. TP53 was downregulated in HPNE cells and predicted to regulate up- and downregulated RNA transcripts, while it was upregulated in PANC-1 cells and predicted to regulate upregulated mRNA. STAT1 was upregulated in PANC-1 cells and downregulated in HPNE cells, but it was predicted to regulate downregulated mRNAs in PANC-1 cells and upregulated mRNAs in HPNE (Figure 6C, Table 2 and Table 3).

### 3.4. Chromosomal Distribution of Differentially Expressed Transcripts Induced by H_2_O_2_

**Figure 7:**
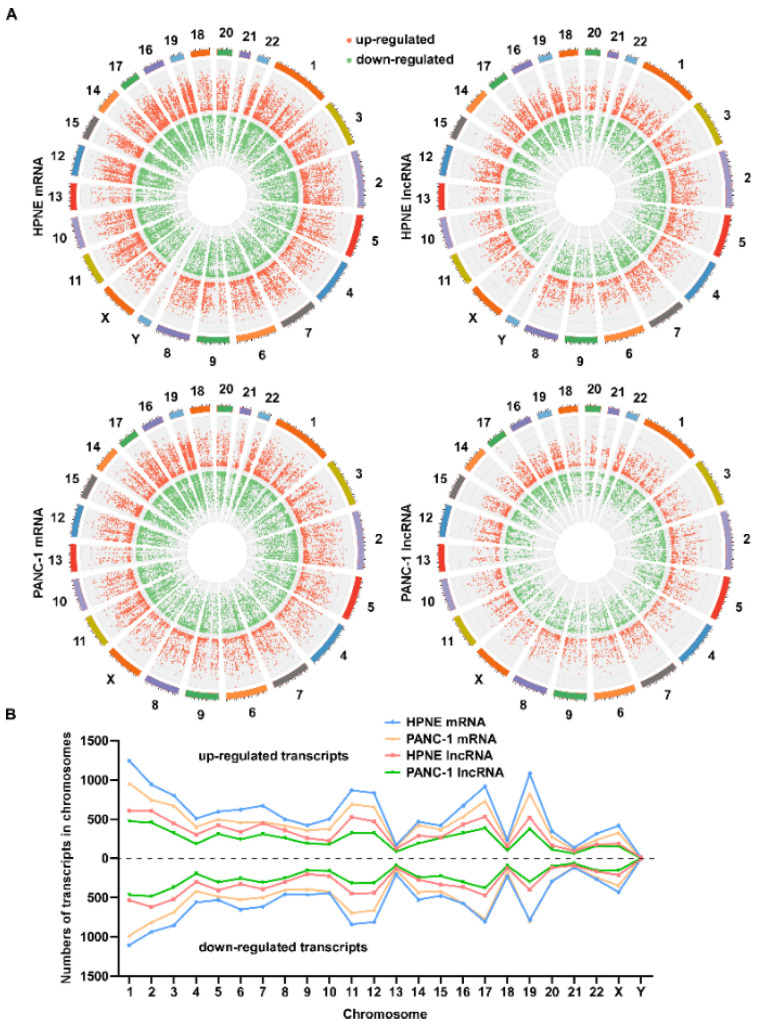
Chromosomal distribution of the differentially expressed transcripts in HPNE and PANC-1 cells treated with H_2_O_2_. (**A**) The chromosomal distribution of differentially expressed mRNA (**left**) and lncRNA (**right**) transcripts upon H_2_O_2_ treatment. Data are shown as Circos diagrams. The red dots represent upregulated transcripts, while the green dots represent downregulated transcripts. Relative height represents log_2_ (fold change). (**B**) Numbers of differentially expressed mRNA and lncRNA transcripts in the indicated chromosomes of HPNE and PANC-1 cells treated with H_2_O_2_. The dots below the dotted line represent the downregulated transcripts, while the top dots represent the upregulated transcripts.

We next examined the distributions of the H_2_O_2_-induced alterations in RNA transcripts among different chromosomes, and we found that the distribution of upregulated and downregulated RNA transcripts on chromosomes appeared correlated with the chromosomal sizes. The numbers and change trends of upregulated or downregulated transcripts in the same chromosomal position were very similar (Figure 7A). The numbers of upregulated and downregulated transcripts in different chromosomes appeared similar, with chromosome 1 containing the most altered transcripts and chromosomes 13, 18, 21, and Y containing fewer altered transcripts (Figure 7B). Similar patterns were observed in differentially expressed lncRNA and mRNA transcripts in HPNE and PANC-1 cells (Figure 7B).

## 4. Discussion

Redox signaling is a fundamental biological process essential for the maintenance of physiological homeostasis and cell survival, especially under stress conditions. Cellular response to ROS stress has been an important area of study by many laboratories, which have collectively gained significant new insights into the regulatory mechanisms and therapeutic implications [5,27,28,29]. Although various aspects of redox signaling by H_2_O_2_ have been reported in the literature, the impacts of H_2_O_2_ on global transcription of mRNA and lncRNA in normal and cancer cells and the respective mechanisms remain to be characterized in detail. The current study is a step toward this direction.

To test the impact of H_2_O_2_ on the expression of mRNA and lncRNA transcripts, it is important to use physiologically relevant concentrations that exert sufficient ROS stress without causing significant cytotoxicity. In our study, two different assays (MTS and flow cytometry analyses) were used to determine the maximum subtoxic concentrations of H_2_O_2_ for normal pancreatic epithelial cells (HPNE, 0.3 mM) and pancreatic cancer cells (PANC-1, 1.0 mM). With these defined conditions, transcriptome sequence was performed to analyze the global changes in mRNA and lncRNA transcripts in response to the H_2_O_2_ exposure. GO and KEGG pathway enrichment analyses unsurprisingly revealed alterations in stress-associated pathways, as well as certain important pathways originally unexpected. Among these changes, it is worth noting that the differentially expressed lncRNAs were enriched in pathways associated with metabolism of RNA, processing of capped intron-containing pre-mRNA, spliceosome, and RNA transport. These novel findings suggest that lncRNAs might play a potentially important role in regulating RNA metabolism and processing in response to ROS stress, thus meriting further study.

A comparison of H_2_O_2_-induced differentially expressed genes in HPNE and PANC-1 cells revealed some significant difference between two cell lines in response to ROS stress. For instance, Figure 1C revealed many transcripts that were upregulated in HPNE cells but downregulated in PANC-1 cells or vice versa. Since HPNE is an immortalized human pancreatic normal epithelial cell line while PANC-1 is a human pancreatic cancer cell line, it is not surprising that they exhibited a significant difference in response to H_2_O_2_ stress. Of note, changes in cell cycle- and DNA repair-associated transcripts were significantly enriched in HPNE cells after H_2_O_2_ treatment, whereas no obvious changes in these transcripts were observed in PANC-1 cells. The enrichment of altered transcripts associated with cell-cycle regulation and DNA repair pathways in HPNE cells is consistent with the notion that normal cells maintain an intact response mechanism to potential DNA damages induced by ROS, whereas such a mechanism appears compromised or even lost in cancer cells, allowing them to proceed with proliferation without proper DNA repair [30]. Mechanistically, the functional status of p53, which plays a major role in sensing DNA damage and cell-cycle regulation, may largely contribute to such different responses observed in the two cell types. Indeed, our results showed that the p53 signaling pathway was activated in HPNE cells, whereas no p53-assocated transcripts were enriched in PANC-1 cells. It should be noted that other signaling pathways might also contribute to the different responses. For instance, our KEGG enrichment analysis showed that the TGF-β signaling pathway was only activated in HPNE cells in response to ROS stress, while the mRNA transcripts associated with the Jak/STAT signaling pathway were mainly seen in PANC-1 cells after H_2_O_2_ treatment. As a double-edged sword, the TGF-β signaling pathway can suppress tumor development by inhibiting cell proliferation and inducing apoptosis, but it can also promote cancer cell invasion and metastasis [31,32,33]. The Jak/STAT pathway is activated in many solid tumor cells and contributes to the malignant properties of cancer cells [34]. The precise roles of TGF-β and Jak/STAT signaling pathways in cellular response to ROS stress require further investigation.

One interesting and somewhat surprising discovery from this study was the findings that the upregulated and downregulated RNA transcripts induced by H_2_O_2_ shared the same sets of promoters. Such seemingly paradoxical findings were consistently observed in mRNA and lncRNA transcripts of both HPNE and PANC-1 cells, as revealed by two transcription factor analysis tools. The PROMO analysis is based on transcript sequencing and, thus, precise for each transcript, while TRRUST prediction is based on gene locus and, thus, more suitable for analysis of *cis*-regulated transcripts. The analyses using both tools consistently revealed that both upregulated and downregulated RNA transcripts shared the same transcription factors in response to H_2_O_2_ stress. These findings seem to suggest that the core regulatory mechanisms in cellular response to ROS stress are highly conserved, but these conserved promoters alone do not determine that final levels of transcriptional output for the individual transcripts. It is likely that the transcriptional activity for the individual genes may be collaboratively determined by the conserved core promoters and the coactivation or inhibition of the transcription factors, which may vary depending on cell types and environmental conditions. Our novel findings reveal the complexity of transcription regulation in response to ROS stress.

Since most differentially expressed lncRNA are extronic, TRRUST was used to predict transcription factors. The results showed that the transcription factors Nrf2, HIF-1α, ATM, NF-κB, and p53 were predicted as promoters of the upregulated lncRNAs, consistent with previously known transcription factors upon ROS stress. Our study suggests that these transcription factors not only regulate protein-coding mRNA expression but also control the expression of lncRNAs.

Our study for the first time revealed a chromosome-dependent transcription regulation in response to H_2_O_2_ stress (Figure 7). There was an overall correlation between H_2_O_2_-induced alterations in expression of mRNA and lncRNA transcripts and the chromosomal length, with notable exceptions. Interestingly, chromosomes 13, 18, 21, and Y exhibited substantially fewer changes in transcripts after H_2_O_2_ treatment, whereas chromosomes 11, 12, 16, 17, 19, and 22 showed more changes in RNA transcripts. Of note, there are some oxidative stress-responsive genes located in these chromosomes. For example, ATM serine/threonine kinase (ATM), and RELA proto-oncogene, NF-κB subunit (RELA) are located on chr11, microsomal glutathione *S*-transferase 1 (MGST1) and thioredoxin reductase 1 (TXNRD1) are located on chr12, NAD(P)H quinone dehydrogenase 1 (NQO1) and metallothionein 1 (MT1 family) are located on chr16, aldehyde dehydrogenase 3 family member A1 (ALDH3A1) is located on chr17, cytochrome P450 family 2 subfamily A member 6 (CYP2A6) is located on chr19, and heme oxygenase 1 (HMOX1) and thioredoxin reductase 2 (TXNRD2) are located on chr22. This might in part explain why these chromosomes exhibited above-average changes in transcripts in response to ROS stress. Moreover, the chromosomal sites of upregulated and downregulated transcripts appeared as “mirror symmetry” (Figure 7B). This finding suggests that the degrees of altered expression of different genes might reflect the local transcriptional activity regulated by complex local factors such as epigenetic modifications of the individual chromosomal segments and local chromosome structural features, which in turn might affect DNA modifications by ROS. It is known that ROS could directly affect DNA by formation of 8-oxo-2′-deoxyguanosine, via hydroxyl radicals (OH•), or by formation of 5-hydroxymethylcytosine (5hmC) [35]. ROS can also indirectly affect DNA methylation at the global or local levels leading to modulation of gene expression [36]. The complex relationship between epigenetic modification and the differential expression of lncRNAs after H_2_O_2_ treatment needs to be further explored.

## 5. Conclusions

In conclusion, our study utilized transcriptome sequencing to determine the global changes in mRNA and lncRNA transcripts induced by H_2_O_2_ in normal pancreatic epithelial and pancreatic cancer cells. Common and differentially expressed transcripts associated with various signaling pathways were characterized. We showed that mRNA and lncRNA largely shared the same sets of transcription factors in response to ROS stress, and the promoters of the upregulated genes were similar to those of the downregulated transcripts, suggesting that these conserved promoters might not be the decisive factors determining the transcription activity of the individual genes; instead, they may need to collaborate with other cofactors/inhibitors to control the final transcriptional output. There was a significant difference between pancreatic cancer and noncancer cells in their response to H_2_O_2_ stress, especially in the transcription of genes involved in cell-cycle regulation and DNA repair. Lastly, our study revealed chromosome-dependent alterations of RNA transcripts in response to H_2_O_2_ stress, likely regulated by local chromosomal factors.

## Figures and Tables

**Table 1:** Description and log *p*-value of KEGG enrichment terms in Figure 3C.

Term	Description	Log P
R-HSA-1640170	Cell cycle	−44.7433
R-HSA-8953854	Metabolism of RNA	−44.3152
R-HSA-194315	Signaling by Rho GTPases	−23.5974
GO:1903047	Mitotic cell-cycle process	−22.8387
WP3888	VEGFA–VEGFR2 signaling pathway	−22.3273
GO:0051603	Proteolysis involved in cellular protein catabolic process	−21.2771
R-HSA-157118	Signaling by NOTCH	−19.9154
R-HSA-3700989	Transcriptional regulation by TP53	−19.7004
R-HSA-5663202	Diseases of signal transduction by growth factor receptors and second messengers	−19.0701
R-HSA-1280215	Cytokine signaling in immune system	−19.0064
GO:0051301	Cell division	−17.9398
R-HSA-109581	Apoptosis	−17.2555
R-HSA-69275	G2/M transition	−16.8881
GO:0097190	Apoptotic signaling pathway	−16.0895
GO:0034976	Response to endoplasmic reticulum stress	−15.357
R-HSA-72203	Processing of capped intron-containing pre-mRNA	−14.5354
R-HSA-109582	Hemostasis	−13.6556
GO:0097435	Supramolecular fiber organization	−13.653
GO:1903827	Regulation of cellular protein localization	−13.3734
ko05203	Viral carcinogenesis	−13.26
R-HSA-1640170	Cell cycle	−44.7433
R-HSA-8953854	Metabolism of RNA	−44.3152

**Table 2:** Transcription factors of significantly changed transcripts in HPNE predicted by TRRUST (Venn diagram in Figure 5B).

Group	Total	Elements
lncRNA-up + lncRNA-down + mRNA-up + mRNA-down	6	STAT3, RELA, TP53, NFKB1, SP1, ESR1
lncRNA-up + mRNA-up + mRNA-down	3	MYCN, MYC, JUN
lncRNA-down + mRNA-up + mRNA-down	2	TFAP2A, AR
lncRNA-up + mRNA-up	7	ETS1, YY1, TP73, RB1, NFE2L2, KLF6, ATM
lncRNA-up + mRNA-down	1	HIF1A
lncRNA-down + mRNA-up	2	BRCA1, SP3
lncRNA-down + mRNA-down	2	SATB1, SOX6
mRNA-up + mRNA-down	11	YBX1, ATF2, ATF3, HDAC1, EGR1, SIRT1, E2F1, NR3C1, KLF4, HDAC2, CREB1
lncRNA-up	1	EP300
lncRNA-down	1	NFIC
mRNA-up	21	CDX2, TCF3, PPARG, SREBF1, ATF4, WT1, STAT1, MYBL2, JUND, POU2F1, POU5F1, BCL6, STAT5A, RARA, ETV6, NFYA, CEBPB, USF2, USF1, SMAD7, NRF1
mRNA-down	11	TWIST2, E2F3, TWIST1, SRF, NF1, MSC, HMGA1, IRF1, EZH2, ETS2, HDAC3
lncRNA-up + lncRNA-down + mRNA-up + mRNA-down	6	STAT3, RELA, TP53, NFKB1, SP1, ESR1
lncRNA-up + mRNA-up + mRNA-down	3	MYCN, MYC, JUN
lncRNA-down + mRNA-up + mRNA-down	2	TFAP2A, AR

**Table 3:** Transcription factors of significantly changed transcripts in PANC-1 predicted by TRRUST (Venn diagram in Figure 5B).

Group	Total	Elements
lncRNA-up + lncRNA-down + mRNA-up + mRNA-down	1	SP1
lncRNA-up + lncRNA-down + mRNA-up	1	MYC
lncRNA-up + mRNA-up	1	E2F1
lncRNA-down + mRNA-down	1	MYCN
mRNA-up + mRNA-down	2	JUN, EZH2
lncRNA-up	4	RELA, NFKB1, SP3, EP300
mRNA-up	6	DDIT3, HIF1A, TP53, SIRT1, AR, ESR1
mRNA-down	5	TWIST2, YY1, TWIST1, STAT1, PPARG

## Data Availability

The data presented in this study are available in the article and Supplementary Materials.

## Supplementary Materials

The following supporting information can be downloaded: https://www.mdpi.com/article/10.3390/antiox11030495/s1, Table S1. TF count analysis by PROMO; Table S2. TF count analysis by TRRUST; Table S3. qPCR primer list. Figure S1. Effect of hydrogen peroxide (H_2_O_2_) on cell viability in normal pancreatic epithelia and pancreatic cancer cells. (A) Immortalized human pancreatic normal epithelia (HPNE) and pancreatic cancer (PANC 1) cells were treated with various concentrations of H_2_O_2_ for various time periods as indicated. Cell viability was measured by MTS assay. *, *p* < 0.01. (B) HPNE and PANC 1 cells were respectively treated with 0.3 mM and 1.0 mM H_2_O_2_ for 12 h. Apoptotic cell death was analyzed by annexin V/PI double staining followed by flow cytometry analysis. Figure S2. Expression of oxidative stress responsive target mRNA and lncRNA in HPNE and PANC 1 cells exposed to sub toxic concentrations of H_2_O_2_. Expression of the transcripts was measured by RNA seq analysis. Figure S3. Distribution of differentially expressed genes associated with “Transcriptional misregulation in cancer” pathway induced by H_2_O_2_. Expression of enriched genes in “Transcriptional misregulation in cancer” pathway in HPNE (left) and PANC 1 cells (right) treated with sub toxic concentrations of H_2_O_2_ (0.3 mM for HPNE and 1.0 mM for PANC 1 cells for 12 h) was measured by transcriptome sequence (A) or by qRT PCR (B). Data are means ± SEM of three separate experiments; two tailed unpaired t test for B; *, *p* < 0.05. Figure S4. Expression of genes associated with “Transcriptional misregulation in cancer” pathway in HPNE and PANC 1 cells treated with H_2_O_2_. Quantitative RT PCR measured Transcriptional misregulation in cancer signal associated genes in HPNE (A) and PANC 1 (B) cells treated with H_2_O_2_ (HPNE: 0.3 mM; PANC 1: 1.0 mM) for 1 h or 12 h as indicated. Data are means ± SEM of three separate experiments; two tailed unpaired t test; *, *p* < 0.05.
