# Supplementary material for: Characterization of H2O2-Induced Alterations in Global Transcription of mRNA and lncRNA
Source: Antioxidants (Basel). 2022 Mar 3;11(3):495. doi: 10.3390/antiox11030495 (PMC8944526; doi:10.3390/antiox11030495)
Supplement: Supplementary file 1 [file antioxidants-11-00495-s001.zip › Supplementary Materials/Supplemental figures and legends.pdf]

Supplemental figure 1

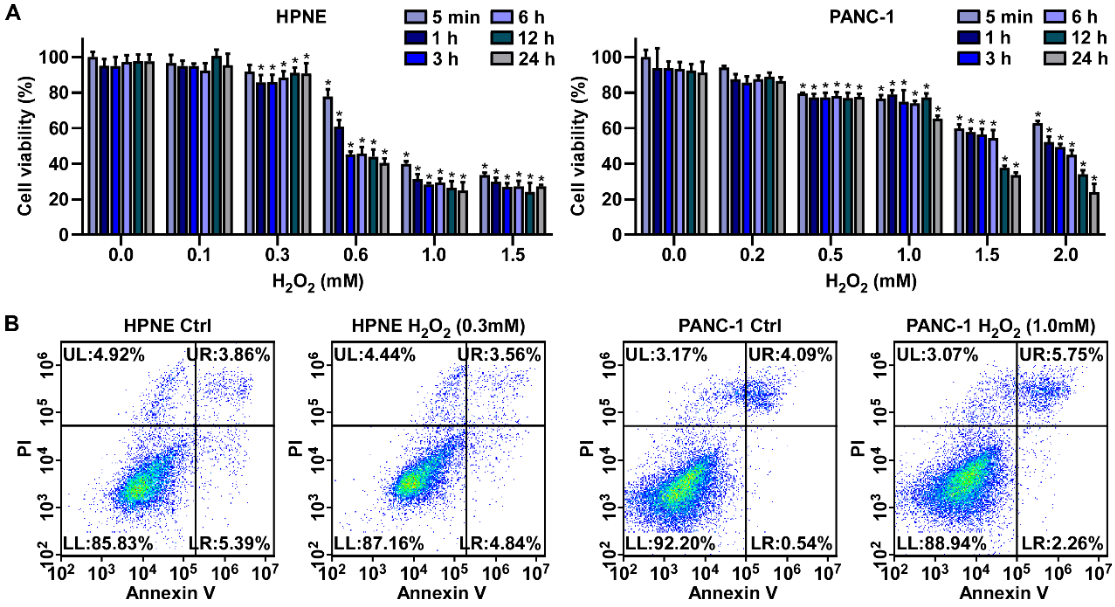

Figure S1. Effect of hydrogen peroxide (H_2O_2) on cell viability in normal pancreatic epithelia and pancreatic cancer cells. (A) Immortalized human pancreatic normal epithelia (HPNE) and pancreatic cancer (PANC-1) cells were treated with various concentrations of H_2O_2 for various time periods as indicated. Cell viability was measured by MTS assay. *, $p<0.01$. (B) HPNE and PANC-1 cells were respectively treated with 0.3 mM and 1.0 mM H_2O_2 for 12 h. Apoptotic cell death was analyzed by annexin-V/PI double staining followed by flow cytometry analysis.

Supplemental figure 2

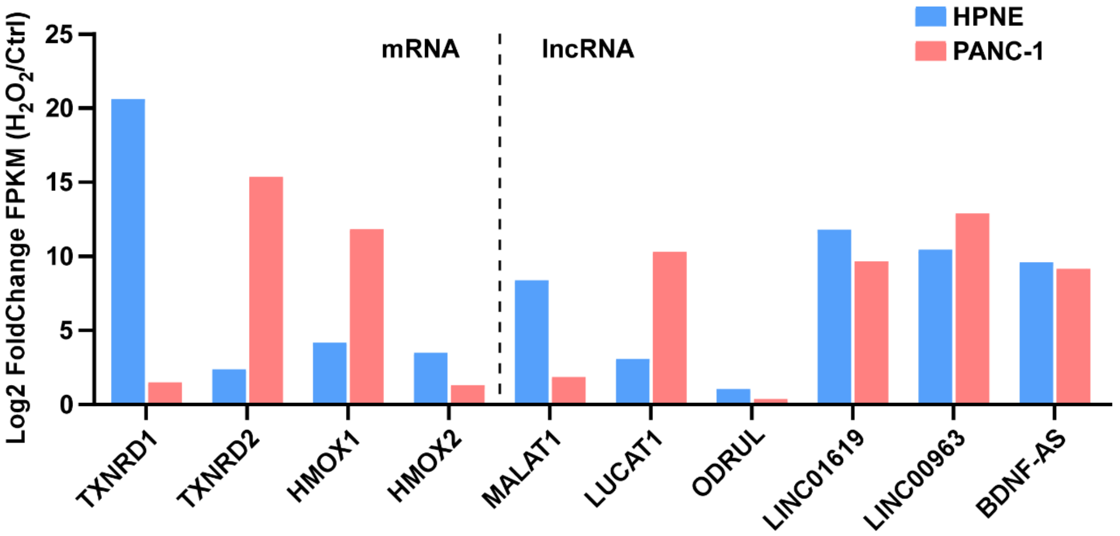

Figure S2. Expression of oxidative stress responsive target mRNA and lncRNA in HPNE and PANC-1 cells exposed to sub-toxic concentrations of H_2O_2. Expression of the transcripts was measured by RNA-seq analysis.

Supplemental figure 3

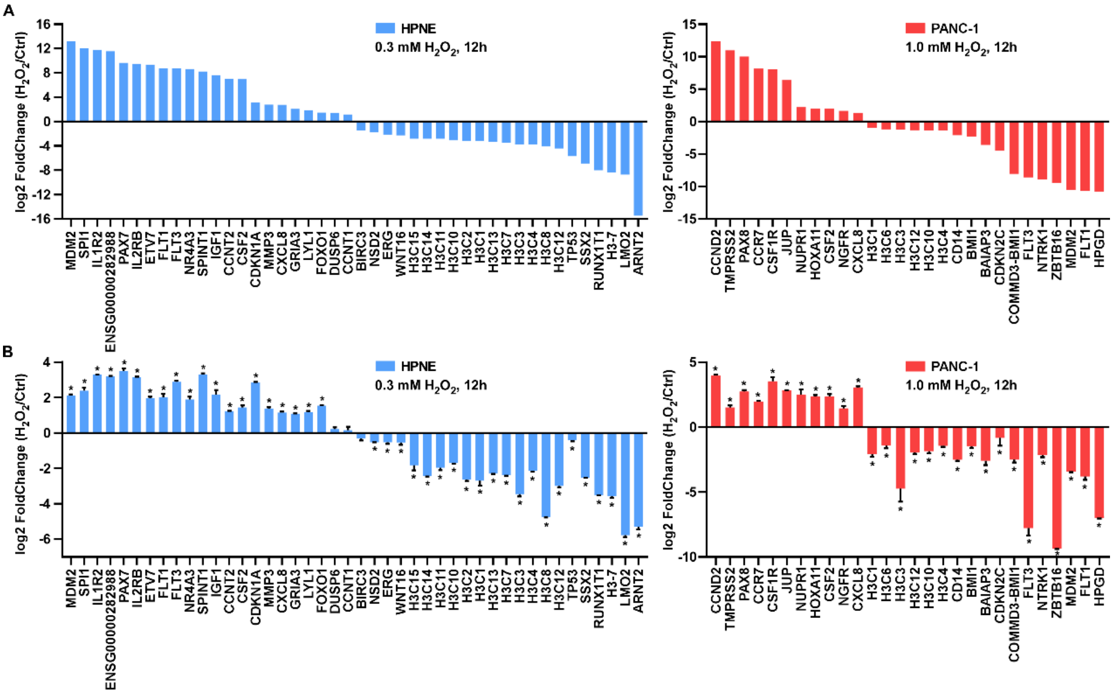

Figure S3. Distribution of differentially expressed genes associated with “Transcriptional misregulation in cancer” pathway induced by H_2O_2. Expression of enriched genes in “Transcriptional misregulation in cancer” pathway in HPNE (left) and PANC-1 cells (right) treated with sub-toxic concentrations of H_2O_2 (0.3 mM for HPNE and 1.0 mM for PANC-1 cells for 12 h) was measured by transcriptome sequence (A) or by qRT-PCR (B). Data are means ± SEM of three separate experiments; two-tailed unpaired t-test for B; *, $p<0.05$.

Supplemental figure 4

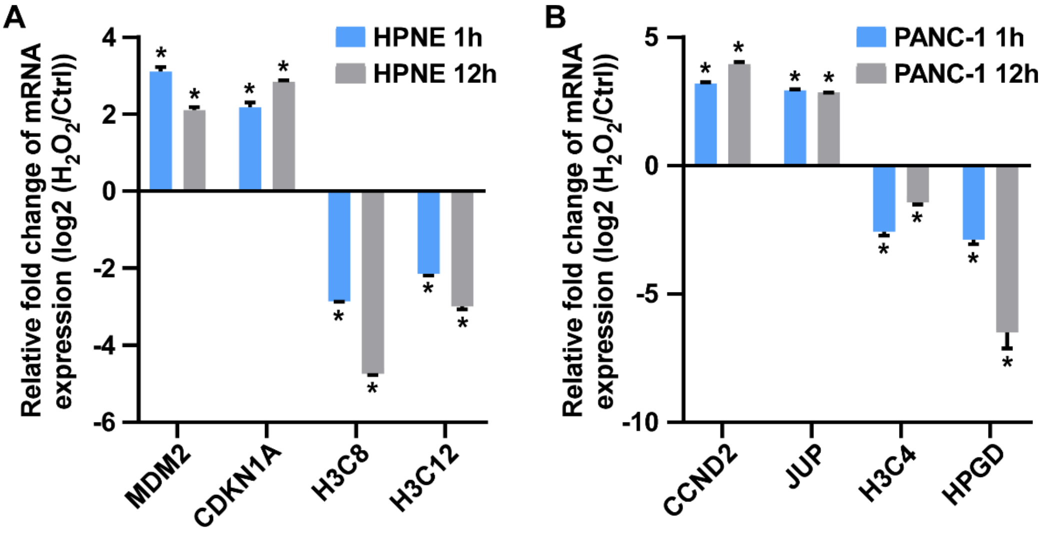

Figure S4. Expression of genes associated with "Transcriptional misregulation in cancer" pathway in HPNE and PANC-1 cells treated with H_2O_2. Quantitative RT-PCR measured Transcriptional misregulation in cancer signal associated genes in HPNE (A) and PANC-1 (B) cells treated with H_2O_2 (HPNE: 0.3 mM; PANC-1: 1.0 mM) for 1 h or 12 h as indicated. Data are means ± SEM of three separate experiments; two-tailed unpaired t-test; *, $p<0.05$.
